# Genetic structuring of remnant forest patches in an endangered medicinal tree in North-western Ethiopia

**DOI:** 10.1186/1471-2156-15-31

**Published:** 2014-03-06

**Authors:** Haile Yineger, Daniel J Schmidt, Jane M Hughes

**Affiliations:** 1 Australian Rivers Institute, Griffith School of Environment, Griffith University, 170 Kessels Road, Nathan QLD 4111, Australia

**Keywords:** Genetic diversity, *Guangua Wereda*, Microsatellite loci, Population genetic structure, *Prunus africana*, Spatial pattern of genetic differentiation

## Abstract

**Background:**

Habitat loss and fragmentation may have detrimental impacts on genetic diversity, population structure and overall viability of tropical trees. The response of tropical trees to fragmentation processes may, however, be species, cohort or region-specific. Here we test the hypothesis that forest fragmentation is associated with lower genetic variability and higher genetic differentiation in adult and seedling populations of *Prunus africana* in North-western Ethiopia. This is a floristically impoverished region where all but a few remnant forest patches have been destroyed, mostly by anthropogenic means.

**Results:**

Genetic diversity (based on allelic richness) was significantly greater in large and less-isolated forest patches as well as in adults than seedlings. Nearly all pairwise *F*_ST_ comparisons showed evidence for significant population genetic differentiation. Mean *F*_ST_ values were significantly greater in seedlings than adults, even after correction for within population diversity, but varied little with patch size or isolation.

**Conclusions:**

Analysis of long-lived adult trees suggests the formerly contiguous forest in North-western Ethiopia probably exhibited strong spatial patterns of genetic structure. This means that protecting a range of patches including small and isolated ones is needed to conserve the extant genetic resources of the valuable forests in this region. However, given the high livelihood dependence of the local community and the high impact of foreign investors on forest resources of this region, in situ conservation efforts alone may not be helpful. Therefore, these efforts should be supported with ex situ gene conservation actions.

## Background

Persistent dependence of human livelihoods on agriculture, tropical rainforests and products has remained the major cause of overharvesting, deforestation, forest degradation and land use alterations in most developing tropical regions [1,2]. Such anthropogenic factors in turn have led to forest loss (the loss of a certain amount of forest habitat from a region) and fragmentation (the sub-division of a formerly large unbroken forest into several forest patches of smaller but variable sizes and spatial configurations) [3]. Thus habitat loss and fragmentation modify landscape composition and spatial configuration [3]. Also, following forest habitat loss and fragmentation, significant modifications in habitat qualities and biochemical conditions may be expected specifically around the edges of remnant forest patches [4]. The changes in landscape composition, spatial configuration, habitat qualities and biochemical conditions may cause negative effects on biotic interactions, reproductive processes, dispersal and species persistence in human modified landscapes [4-11]. Fragmentation may also disrupt gene flow among populations and ultimately contribute to the formation of small and isolated populations, which are more vulnerable to extinction due to demographic, genetic and environmental stochastic events [10,12].

Conservation genetics studies are expected to provide information that enables a better understanding of the ways in which human disturbance and fragmentation processes affect population viability in fragmented landscapes and this may assist in devising more appropriate management options [11,13]. Small and isolated populations in fragmented landscapes may experience reduced genetic connectivity, high rates of random genetic drift and elevated inbreeding [13-15]. These may increase genetic differentiation among remnant populations and may erode the populations’ genetic variability. The erosion in genetic variation may in turn reduce individual fitness and a population’s ability to adapt to changing environmental conditions [14,16]. These genetic effects of habitat loss and fragmentation could be unraveled by conducting species-specific empirical investigations in appropriate systems [17].

However, empirical research has documented contradictory genetic effects of habitat loss and fragmentation especially on long-lived tree species [18]. That is to say, some studies have shown adverse effects of habitat loss and fragmentation on the genetic diversity and structure of various tree species [19-22] whereas others did not find genetic effects of forest fragmentation [23-25]. The absence of genetic effects of fragmentation among long-lived tree species is most likely due to the recent history of human disturbance and fragmentation events whose genetic effects will only be reflected after multiple generations which might not have been accounted for by some researchers [26]. In spite of this, the historical and contemporary effects of fragmentation may be fairly assessed and compared by working on different life history stages of trees such as fruiting adults and early stage seedlings [27]. Genetic variation among adults may reflect pre or historical fragmentation effects whereas contemporary effects will be observable among seedlings. Accordingly, fruiting adults are expected to have higher genetic variability and lower genetic differentiation than seedlings [20,27,28]. Application of highly informative molecular tools such as microsatellites [29] may reveal these expected patterns.

Nevertheless, genetic effects of fragmentation may still remain undetected or absent depending on a number of other contexts. First, capacity of some tree species for extensive gene flow by pollen or seed can buffer a population against genetic effects of fragmentation [23,30]. Second, recent debates [31] argued that enhanced gene flow can be observed in more fragmented and disturbed landscapes in at least two ways: 1) if dispersal vectors are not strictly dependent on intact forest habitats, a physical opening of canopies arising from disturbance and fragmentation may allow them to move freely and cover longer distances; 2) human disturbance and fragmentation cause reduction in density of potential mates at a site. As a result, there will be less competition between local and immigrant propaguales thus likely facilitating better gene flow.

The remnant forest patches of Awi Zone in North-western Ethiopia are mainly dominated by *Prunus africana, Albizia schimperiana, Albizia gumifera and Celtis africana*. According to the recent natural vegetation map of Eastern Africa [32], nearly all of these plant species potentially occur or are cited as indicator or characteristic species of Afromontane rainforests in Ethiopia. In recent years, the previously large and continuous rainforests of this region have been lost because of continued human exploitation and habitat fragmentation. North-western Ethiopia harbours several forest patches of this currently rare rainforest type. The surrounding matrix consists of open agricultural land with a few remnant tree species such as *Prunus africana* and *Albizia schimperiana*. The remnant forest patches are under heavy pressure due to uncontrolled grazing and illegal logging by the local inhabitants. The remnant forest patches are thus highly threatened due to human disturbance and progressive subdivision. Conducting empirical investigations on the genetic effects of human disturbance and habitat fragmentation on representative taxa may help to formulate valid recommendations for the conservation and restoration of these fragile ecosystems.

In this study, we chose *Prunus africana* (Hook.f.) Kalkman (Rosaceae) as a model species to assess the impacts of habitat loss and fragmentation in Awi Zone of North-western Ethiopia. *P. africana* is a long-lived monoecious tree species that has been listed as Endangered on Appendix II of CITES since 1995 and as Vulnerable in the IUCN Red List of Threatened Species since 1998 [33] but is locally abundant in the study region. The bark of *P. africana* is highly valued in the international medicinal plant trade as it is the major source of an extract used to treat urinary disorders, prostate gland hypertrophy and benign prostatic hyperplasia [34]. Because of this, either the bark or partially processed bark extract has been heavily and unsustainably exported from Africa (especially Cameroon, Madagascar, and Kenya) to Europe causing concerns for its conservation and sustainable utilization [35]. In Kenya, a fragmentation genetics study of *P. africana* at the Kagamega forest revealed significantly lower genetic variability among seedlings than adults [28]. However, data are lacking on genetic variability and the extent of population differentiation among the remnant forest patches of North-western Ethiopia. Conserving and rehabilitating these otherwise vanishing forest genetic resources require more region-specific and detailed studies. So, our findings, which are based on several new analyses than used in Farwig et al. [28], are worth to inform more appropriate management and conservation actions.

We used seven polymorphic microsatellite loci to test the hypothesis that recent and ongoing forest fragmentation explains patterns of genetic variation in remnant forest patches. Under this scenario, the relative magnitude of genetic differentiation among remnant patches would be associated with their degree of fragmentation. Accordingly, we predict a) less variation in smaller relative to larger forest patches; b) more evidence of bottleneck effects in smaller than larger patches; and c) more structure in seedlings than adults. Alternatively, if recent and ongoing fragmentation does not explain patterns of genetic variation in remnant patches, we predict either panmixia across the study area, or an isolation by distance (IBD) effect associated with historical structuring of the forest that predates its fragmentation.

## Results

### Linkage and Hardy-Weinberg equilibrium

Analysis of data in Micro-checker revealed the potential occurrence of null alleles in one of the 8 loci. We analyzed data at both 8 and 7 loci and found some variation in statistical outputs between the two. As a result, we decided to omit the locus suspected to contain null alleles (EMPAS01) from further analyses. No significant linkage disequilibrium was detected for the various locus pair combinations consistently across all populations indicating independence of loci used. Significant Hardy-Weinberg disequilibria were detected for some locus-population pairs for locus EMPA001 and PS12A02 even after sequential Bonferroni corrections. However, these loci did not consistently deviate from Hardy-Weinberg equilibrium for all populations. We retained these loci for analyses because results were robust regardless of their exclusion or inclusion.

### Genetic variability of remnant forest patches

All the 7 loci were highly polymorphic with the number of alleles sampled per locus ranging from 7 to 29 in adults and 5 to 24 in seedlings. The total number of alleles sampled at the 7 loci over all forest patches was 129, 123 and 96 for the combined data set, adults and seedlings, respectively. The mean number of alleles sampled per patch across 7 loci ranged from 5.6 to 11 (overall mean = 8.82) in adults and 5 to 9.1 (overall mean = 7.63) in seedlings. The difference between these overall means was significantly different from zero (Table 1). The overall mean of expected heterozygosity (H_S_) per patch across 7 loci was significantly greater in adults (H_S_ = 0.77) than seedlings (H_S_ = 0.71). Similarly, the overall mean of allelic richness per patch was significantly greater in adults (5.96) than seedlings (4.85) (Table 1). However, the overall mean of *F*_IS_ per patch did not differ significantly between adult and seedling populations (Table 1).

**Table 1:** **Comparison of genetic diversity estimates (averaged across 7 loci) between cohorts of *Prunus africana *** **populations (Note: Allelic richness per locus and population was based on a minimum sample size of 7 and 6 diploid individuals, respectively in adults and seedlings**

**Population**	**n**		**Allelic richness**		**H**_**S**_		***F***_**IS**_		**MNA**	
	**Adult**	**Seedling**	**Adult**	**Seedling**	**Adult**	**Seedling**	**Adult**	**Seedling**	**Adult**	**Seedling**
Bradi (L, C)	26	36	6.567	4.987	0.817	0.746	-0.010	-0.176	10.000	9.143
DarabaSigsi (L, C)	29	20	6.372	4.829	0.793	0.713	0.171	0.082	9.714	7.000
Demba (S, C)	25	30	6.045	4.479	0.758	0.716	0.006	-0.332	9.571	6.857
Dishi (S, I)	33	49	5.917	5.239	0.767	0.738	0.070	-0.002	9.000	9.571
Kambo (L, C)	44	28	6.185	5.442	0.768	0.739	0.096	-0.006	11.000	8.286
Metin (S, I)	12	10	5.209	4.338	0.732	0.654	0.027	-0.026	5.571	5.000
Temcha (S, I)	24	30	5.623	4.564	0.757	0.692	-0.06	-0.038	8.143	7.143
Wonse (L, I)	17	29	5.733	4.938	0.76	0.711	-0.24	0.001	7.571	8.000
Paired t-test (Adult-Seedling) p-value			<.0001		0.0001		0.2694		0.0433	
t-value			7.89		7.54		1.2		2.46	
DF			7		7		7		7	

Allelic richness values in this table represent mean values across loci at each population. Hs = gene diversity; *F*_IS_ = inbreeding coefficient; MNA = mean number of alleles; n = sample size; L = large; C = less-isolated).

For the overall data set, average allelic richness per forest patch was significantly (t-test: t = 3.14, DF = 6, *P* = 0.0201) greater for large (range: 7.91 to 8.67, mean = 8.38) than small (range: 6.78 to 7.89, mean = 7.44) forest patch groups (Table 2 and Additional file 1). Comparison of average allelic richness per patch also revealed significantly greater diversity for the large than small forest patches. Similarly, mean allelic richness was significantly (t-test: t = 2.57, DF = 6, *P* = 0.0425) greater for less-isolated (range: 7.76 to 8.67, mean = 8.34) than isolated (range: 6.78 to 7.91, mean = 7.48) forest patches (Table 2 and Additional file 1). Further, linear regression showed a positive relationship between allelic richness and patch size and a negative relationship with patch isolation (Additional file 2). These linear regression tests were, however, non-significant.

**Table 2:** **Variation in mean allelic richness between large and small, and less-isolated and isolated populations of *Prunus africana *** **for the three data sets (Note: Allelic richness per locus and population was based on a minimum sample size of 7 and 6 diploid individuals, respectively in adults and seedlings)**

**Data set**	**Population group**	**Mean allelic richness**	**Std Err**	**Population group comparison**	***p*-value**	**t-value**	**DF**
Combined	Large	8.3755	0.1638	Large vs small	0.0201	3.14	6
	Small	7.4390	0.2495				
	Less-isolated	8.3370	0.2007	Less-isolated vs isolated	0.0425	2.57	6
	Isolated	7.4775	0.2680				
Adults	Large	6.2143	0.1784	Large vs small	0.0919	2.00	6
	Small	5.6985	0.1855				
	Less-isolated	6.2923	0.1135	Less-isolated vs isolated	0.0118	3.57	6
	Isolated	5.6205	0.1500				
Seedlings	Large	5.0490	0.1351	Large vs small	0.1539	1.63	6
	Small	4.6550	0.2002				
	Less-isolated	4.9343	0.1998	Less-isolated vs isolated	0.5813	0.58	6
	Isolated	4.7698	0.1994				

The Wilcoxon’s signed rank test for mutation-drift equilibrium under TPM did not reveal significant population demographic bottlenecks or recent changes in effective population sizes of remnant forest patches (Additional file 3). In these bottleneck tests, distribution of allele frequencies followed a normal mode shift for all populations (data not shown).

### Population differentiation and genetic structure

**Figure 1:**
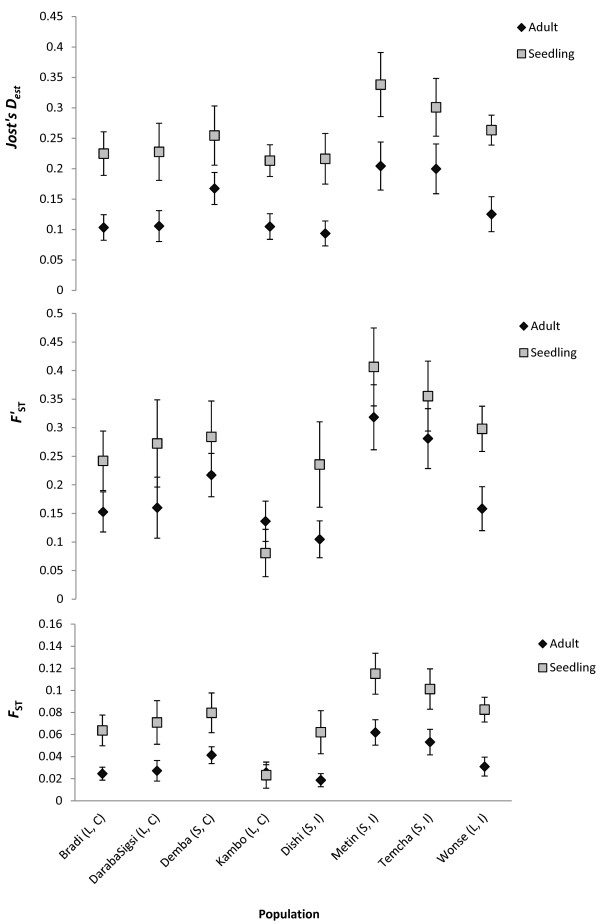
**Comparison of Jost’s D**_**est**_**, *F*’**_**ST **_**and *F***_**ST **_**between adult and seedling populations of *Prunus africana *** **(Note: error bars show the standard error of the mean; L = large; C = less-isolated).**

**Figure 2:**
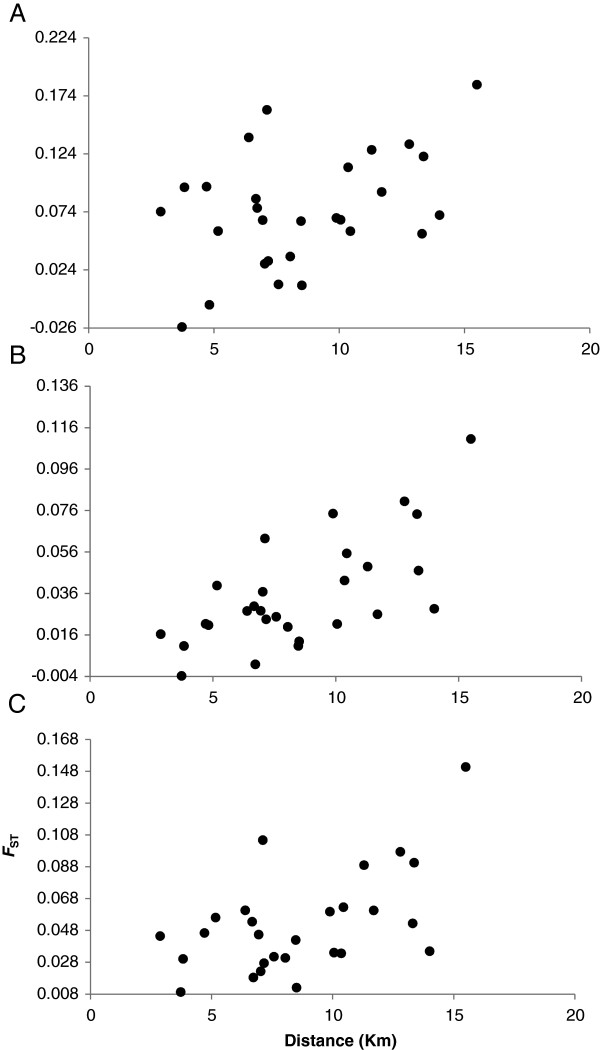
Correlation of genetic vs geographical distances (significance testing at 100000 permutations for Mantel test) (Note: A = Seedlings, B = Adults, C = Overall).

All pairwise *F*_ST_ estimates were significantly higher than random for the overall data set. Furthermore, most pairwise *F*_ST_ comparisons showed evidence for significant population genetic differentiation in both adults and seedlings. Average population-specific *F*_ST_ values were significantly (paired t-test, Adult-Seedling, *p* = 0.0004, t-value = -6.27, DF = 7) greater for seedlings (*F*_ST_ = 0.075) than adults (*F*_ST_ = 0.035) (Figure 1), but varied little (test results not shown) with patch size (large, *F*_ST_ = 0.038 vs small, *F*_ST_ = 0.062) or patch isolation (isolated, *F*_ST_ = 0.063 vs less-isolated, *F*_ST_ = 0.038). Even so, linear regression, though non-significant, revealed a positive relationship between *F*_ST_ and patch isolation and an inverse relationship with patch size (Additional file 2). The trends of average population-specific *F*_ST_ comparisons between the seedling and adult populations were similar to corrections using the standardized *F*_ST_ and Jost’s D_est_ (Figure 1). The Mantel test for correlation between genetic and spatial distances was significant (r = 0.569, *p* = 0.0025) for the combined data set demonstrating an isolation by distance (IBD) effect (Figure 2). A significant pattern of IBD was also detected in adults (r = 0.68, *p* = 0.0003) and seedlings (r = 0.43, *p* = 0.0214) (Table 3). Results from simple and partial Mantel tests showed patch size and nearest distance (between edges of patches) were not significantly correlated with genetic variance after accounting for geographic distance (Table 3).

**Table 3:** **Simple and partial Mantel tests (100000 permutations) to assess effects of fragmentation variables on spatial patterns of genetic differentiation in *Prunus africana *** **populations**

**Simple Mantel test**	**Variable**	**Combined data set**		**Adults**		**Seedlings**	
		***P*-value**	**r**	***P*-value**	**r**	***P*-value**	**r**
	GD	0.0025	0.5692	0.0003	0.6751	0.0214	0.4286
	PS	0.9908	-0.4488	0.9207	-0.3299	0.9861	-0.3960
	ND	0.7387	-0.2068	0.7161	-0.1813	0.9412	-0.4372
Partial Mantel test							
GD constant	PS	0.9881	-0.4446	0.9146	-0.3229	0.9863	-0.4062
	ND	0.7132	-0.1955	0.6899	-0.1672	0.9420	-0.4461
PS constant	GD	0.0049	0.5510	0.0006	0.6632	0.0338	0.3887
	ND	0.6024	-0.1180	0.5613	-0.0675	0.9163	-0.3986
ND constant	GD	0.0018	0.5976	0.0003	0.6886	0.0213	0.4326
	PS	0.9971	-0.4905	0.9501	-0.3730	0.9872	-0.4005

Note: GD = |pairwise geographical distance between centers of patches|; PS = |pairwise patch size difference|; ND = |pairwise nearest distance difference between edges of patches|.

The GESTE analysis for pairs of environmental variables against population-specific *F*_ST_ values showed the highest posterior probabilities for geographical distance (from centers of patches) and nearest patch distance (edge to edge) consistently in the combined data set, adults and seedlings (Table 4). The next highest posterior probability was for the pair nearest patch distance and patch size and this was observed only in the seedling cohort (Table 4).

**Table 4:** P**osterior probabilities of models supported by the pairwise GESTE analyses of fragmentation variables against population-specific *F***_**ST**_

**Data set**	**Variables tested**	**Highest probability model (HPM)**	**Posterior probability of HPM**	**Posterior probability of individual factors**
Combined	ND, PS, ND*PS	Constant	0.211	P(ND) = 0.386
				P(PS) = 0.381
				P(ND*PS) = 0.205
	PS, GD, PS*GD	Constant	0.213	P(PS) = 0.399
				P(GD) = 0.393
				P(PS*GD) = 0.194
	ND, GD, ND*GD	Constant, ND*GD, GD, ND	0.272	P(ND) = 0.360
				P(GD) = 0.356
				P(ND*GD) = 0.272
Adults	ND, PS, ND*PS	Constant	0.209	P(ND) = 0.395
				P(PS) = 0.384
				P(ND*PS) = 0.205
	PS, GD, PS*GD	Constant	0.205	P(PS) = 0.405
				P(GD) = 0.402
				P(PS*GD) = 0.189
	ND, GD, ND*GD	Constant, ND*GD, GD, ND	0.276	P(ND) = 0.348
				P(GD) = 0.360
				P(ND*GD) = 0.276
Seedlings	ND, PS, ND*PS	Constant, ND*PS, ND, PS	0.217	P(ND) = 0.392
				P(PS) = 0.388
				P(ND*PS) = 0.217
	PS, GD, PS*GD	Constant, PS	0.204	P(PS) = 0.399
				P(GD) = 0.396
				P(PS*GD) = 0.195
	ND, GD, ND*GD	Constant, ND*GD, GD, ND	0.268	P(ND) = 0.357
				P(GD) = 0.359
				P(ND*GD) = 0.268

Note: ND = Edge to edge distance from the nearest neighbor patch; PS = Patch size or area; GD = Mean of the pairwise center to center patch distance.

Analysis of genetic data for first generation migrant detection revealed limitation in propagule dispersal. Only up to 10% of individual seedlings were migrants. Also, first generation migrant detection for the adult populations showed that less than 8% of adult individuals were migrants (Additional file 4).

## Discussion

Remnant patches of *Prunus africana* in NW Ethiopia exhibit a pattern of higher genetic diversity in larger and less isolated patches relative to smaller and more isolated patches. This trend is expected under conservation genetics theory [36]. Remarkably, analysis of population genetic structure among patches revealed possible limitation in both pollen and seed dispersal that is likely to reflect the pre-fragmentation state of formerly vast and contiguous forests of the study region. Reduced gene flow alone may not, however, explain the strong genetic structuring [37,38]. Our results also showed that the extent of genetic differentiation was more than twice in seedlings than adults. This finding is, however, different from that of Farwig et al. [28] who reported only a very slightly greater genetic differentiation in seedlings than adults. Also, while Farwig et al. [28] did not find significant correlations between spatial and genetic distances in adults and seedlings, our results showed significant correlations in both cohorts. The differences between findings of the two studies might be attributed to several factors. First, our sampling design was slightly different, i.e., it involved sampling adults across transect lines and up to six seedlings per adult tree, whereas Farwig et al. [28] sampled two seedlings per adult tree. Second, there might be variation in the degree of polymorphism in the different sets of marker loci used; only 2 loci are shared between the two studies. Third, regional differences with respect to various ecological attributes such as edaphic and climatic factors, intensity of logging, and vegetation condition that can determine distribution and persistence of gene dispersal agents might explain the observed differences.

In addition to the findings of Farwig et al. [28], our results contribute to understanding of population structure in *P. africana* by 1) providing evidence for spatial patterns of genetic structuring and potential causes after controlling for correlating factors; 2) showing no evidence for recent reduction in effective population sizes; and 3) accounting for effects of genetic diversity when evaluating genetic differentiation.

### Comparison of genetic diversity between seedling and adult cohorts

Despite the relatively larger sample size of seedlings, we found a significantly lower number of alleles, expected heterozygosity and allelic richness in the seedling than adult populations. The larger homozygosity among seedlings appears to be a result of mating amongst close relatives (spatial structure) as is common in trees, but this may decay via demographic thinning or selection against homozygotes. In addition, the sampling was performed on sets of seedlings gathered around mother trees. Therefore seedlings are likely related and share some alleles, thus reducing allelic richness and heterozygosity. Also, gene flow might have changed due to the changed spatial distribution of adult trees following human disturbance and fragmentation [39-41]. Thus reduced gene flow among remnant patches might have contributed to the production of seedlings with lower genetic diversity. However, the observed significant difference in genetic diversity between the two cohorts might not be ecologically relevant if selection favours heterozygote survival. A more likely explanation is that the adult cohort represents overlapping generations while the juvenile cohort represents only a few reproductive events. Thus the adult cohort could be expected to possess higher genetic variability and this variation might not be adequately represented in the seedling cohort.

Although there are no previous data on the disturbance and fragmentation history of the study region, photographic evidence taken during field observations demonstrate the occurrence of extensive logging and felling of age-old *P. africana* trees in the remnant forest patches (see Additional file 5). As already described by Farwig et al. [28] and Hensen et al. [42], such destruction and removal of reproductive adult trees might result in fecundity variance [43], reduced effective population size and limited gene flow among the remnant populations and as a result might have caused reduced genetic diversity in the seedling cohort. These findings are in agreement with our prediction that the younger cohort exhibits genetic effects of fragmentation more strongly than the adult cohort. Results regarding the reduced genetic diversity in the juvenile cohort are also comparable to empirical research on populations of the same species in other regions [28] and other long-lived tree species such as *Polylepis incana*[42], *Vateriopsis seychellarum*[20], *Swietenia humilis*[7], *Quercus ilex*[22] and *Elaeocarpus grandis*[44].

### Comparison of genetic diversity between small and large remnant forest patches

Regression analyses of genetic diversity with both patch size and isolation were non-significant. Despite these, the observed patterns conformed to our expectations that reduction in patch size and increase in isolation would cause lower genetic diversity in *P. africana* populations. This finding is in line with a simulation study [13], which showed that genetic diversity may decline following post-fragmentation reductions in population sizes after several generations. Additionally, based on the patch size and isolation category comparisons for the overall data set, large and less-isolated patches had significantly higher genetic diversity than small and isolated patches. Similar results have been reported for other species [7]. A possible explanation for our findings, however, could be that genetic variability between patches might reflect the legacy of pre-fragmentation genetic structuring (see next section below). In adults, however, only patch isolation was significant. Unexpectedly, both patch size and isolation (based on nearest neighbor distance) did not show significant differences in the seedling cohort but the trend still conformed to our expectation thus depicting very recent signatures of fragmentation genetic effects. As described in [6], such results might also arise from insufficient statistical power for comparisons of the remnant populations due to the relatively small sample sizes.

According to recent range-wide studies on the origin and historic migration routes of *P. africana*[45,46], populations of the species in Ethiopia might be more genetically diverse than populations in other regions. In the current study, however, we found mixed results when comparing different genetic diversity parameters between populations in NW Ethiopia and Kakamega forest of Kenya [28]. For instance, the total number of alleles and the mean number of alleles per population was greater for the NW Ethiopian populations than those in Kakamega forest. In contrast, the mean expected heterozygosity per population was slightly lower in the NW Ethiopian than Kakamega populations. These differences can be attributed to differences in the quality of studied forests between the two regions, the size of populations and the levels of gene flow and connectivity between populations in each region. This comparison, however, should be viewed with caution because Farwig et al. [28] used a different set of loci (only two loci are shared).

### Assessment of population bottlenecks among the remnant forest patches

There was no sign of severe reductions in effective population sizes (N_e_) in the past, 2 N_e_ – 4 N_e_ generations. Given the ongoing severe deforestation and fragmentation processes, we might have expected to detect signs of bottlenecks in the remnant populations, especially the more isolated ones. This finding, however, may be additional evidence for the very recent human disturbance and that fragmentation ‘may not yet have left a population genetic signature’ [6] in the study region. Our informal discussion with some of the elderly local people also showed that fragmentation in NW Ethiopia in particular Guangua Wereda of Awi Zone was a very recent phenomenon (i.e., less than 100 years). According to the elderly local people, the most devastating forest destruction and fragmentation in this region occurred especially in the past 15-20 years. This reflects the absence of law enforcement exercises and also the lack of concerns and attention for conservation of forest resources of this region. To some extent, the failure to detect recent genetic bottlenecks might reflect the use of a relatively small number of loci (n = 7) as the recommended number of loci is at least 10, although the Wilcoxon test has been shown to be sufficiently powerful with as few as 4 loci [47-49]. Also, the power to detect bottlenecks is often low when the decline is not instantaneous or when not many generations have passed. A similar decline occurred in butternut, a North American tree [50], but there was little power to detect the bottleneck due to the low number of markers and shape of decline [51].

### Population genetic structure and spatial patterns of differentiation

Pairwise *F*_ST_ values between populations revealed significant genetic differentiation suggesting restricted gene flow or limited propagule (pollen and seed) dispersal of *P. africana* in NW Ethiopia. The limitation in propagule dispersal was supported by our first-generation migrant detection analysis in Geneclass2 [52], which demonstrated that only up to 10% of individual seedlings in each population were migrants to the respective sampled patches. As pollen and seed dispersal in *P. africana* are facilitated by animal dispersal vectors, possible disturbance and fragmentation effects on mobility, abundance and survival of these agents might have caused the limitation in pollen and seed dispersal [53]. In particular, the high genetic structuring might have resulted from limited spatial patterns of seed dispersal followed by seedling establishment and recruitment filters such as early seedling mortality [37] and selective logging of adult recruits [54]. In addition to the possibility of altered gene flow patterns, other demographic characteristics (e.g., differences in effective population sizes, divergence times and colonization history) as well as evolutionary processes such as mutation and genetic drift might have caused the significant genetic differentiation between populations [37,38].

Average population-specific *F*_ST_ values did not significantly vary with patch size or isolation but observed patterns matched theoretical expectations perhaps implying slightly better gene flow rates during early or pre-fragmentation stages but reduced gene flow after severe fragmentation. These results could also be attributed to the lack of statistical power due to the relatively small sample sizes of patch isolation and patch size groups or the limited number of marker loci, or may indicate little or very recent impacts of fragmentation on population differentiation of *P. africana* in NW Ethiopia.

The mean population-specific *F*_ST_ values of the seedling cohort were significantly greater than the adult. One explanation could be that the seedling cohort was more genetically isolated than the adult as a result of recent human disturbance and habitat fragmentation. However, it may also reflect the differences in genetic diversity between the two cohorts. As *F*_ST_ measures within versus between population diversity, an increase in within population diversity will automatically reduce the *F*_ST_ value. The use of standardized *F*_ST_ (*F’*_ST_) and newer estimates of genetic distance, Jost’s D_est_[55,56], may be expected to account for this effect. The pattern of genetic differentiation between the seedling and adult cohort, however, remained similar even after using these corrections.

We would have expected a non isolation by distance effect, with only the fragmentation variables affecting the spatial patterns of genetic differentiation among the populations. However, both simple and partial Mantel tests revealed a strong effect of distance on genetic differentiation particularly in the adult populations. This pattern almost certainly predated forest fragmentation in this region, thus indicating limitation in both pollen and seed dispersal even in the previously vast and contiguous forests of the study region. Our first-generation migrant detection analysis for the adult populations also supports this prediction. The migrant analysis revealed that less than 8% of sampled adult individuals from a patch were migrants. As the main seed dispersal agents of *P. africana* are thought to be highly mobile animals such as frugivorous birds and mammals [57], better gene flow or seed dispersal among remnant populations would have been anticipated, of course, assuming a negligible impact of fragmentation on dispersers. Our work on bird community and functional trait analysis in the current study region (Yineger and Hughes, *in review*), however, demonstrated a significant impact of fragmentation on the diversity and abundance of avian frugivores. Such impacts of fragmentation on potential dispersers could limit propagule dispersal [58] and hence might be linked to the possibility of altered gene flow patterns. Estimating gene flow by pollen and seed might help to understand the species response to fragmentation. Also, mating system might be influenced by fragmentation (e.g. increased selfing with increased isolation) [26,59] and increased selfing might increase *F*_ST_[13]. We are currently working to understand fragmentation impacts on the mating system and probability of reproduction of *P. africana* and are hoping to communicate this in a separate paper.

## Conclusions

Present day forest patches of Guangua Wereda in NW Ethiopia are remnants after loss of habitat from a previously large and contiguous forest. Analysis of long-lived adult trees suggests the former forest probably exhibited strong spatial patterns of genetic structure (IBD). This means that protecting a range of patches including small and isolated ones is needed to conserve genetic diversity in this degraded landscape. This should, however, be supplemented with conserving trees growing in the surrounding farmland and pasture because matrix trees have high reproductive output and potential to act as links between populations [60]. Nevertheless, given the high livelihood dependence of the local poor community and the high impact of foreign investors on forest resources of this region, in situ conservation efforts alone may not be helpful. Therefore, these efforts should be supported with ex situ gene conservation actions. Of course, it is known that seeds of *P. africana* are partially recalcitrant, which means that seeds may not be stored in gene banks for longer periods. Field gene banks appear better options when considering ex situ conservation. However, this by itself may not be easy to implement. Also it may not adequately represent the extant wild genetic variation of the species. Therefore, more emphasis should be given to the in situ conservation while increasing political commitment and conservation awareness among the local people.

## Methods

### Study species

*Prunus africana* is an endangered evergreen Afromontane medicinal tree species endemic to sub-Saharan Africa and nearby islands [61]. No reliable information is available regarding the longevity of *Prunus africana*[62] but we guess about 200 years based on our field observation of stumps and girth of old trees in NW Ethiopia. It grows to a height of 40 m and diameter of 1 m with elliptic-oblong leathery leaves, small flowers with creamy white petals, transversely ellipsoid dark reddish brown fruits, and dark brown to grey longitudinally fissured or scaly bark [63]. The flowering season is mainly between November and February with potential intermittent flowering that is observed all year round in some localities. Pollination of *P. africana* is assisted by insects and fruits start to develop within 4-6 months after pollination (ICRAF, undated). Fruit ripening usually occurs between September and November but in Kakamega forest of Kenya, Farwig et al. [57] observed individual trees with ripened fruits in October, December and March. The peak fruiting season in the current study area extends from February to April. Ripened fruits are dispersed by a number of bird species and monkeys [57]. Seeds of *P. africana* seem to be recalcitrant. Fresh seeds can readily germinate under shady conditions [34]. The species is, however, light demanding beyond the sapling stage [64].

### Study sites and sampling design

The study sites were located in the fragmented landscapes of Awi Zone, Gojjam, NW Ethiopia, at about 10°45’-11°04’ N and 36°25’-36°48’ E. This is a floristically impoverished region where all but a few remnant forest patches have been destroyed, mostly by anthropogenic means. The major rainy season extends from June to October. Mean annual rainfall is about 1500 mm and mean annual temperature ranges between 19°C and 30°C. Elevation of remnant forest patches ranges between approximately 1750 and 2390 m. Matrix surrounding remnant patches is dominated by agricultural land cultivated in various shifts for millet, corn, tef (*Eragrostis tef*), wheat, barley and rarely beans. The matrix also has some range lands or pastures. Both the matrix of the pasture and agricultural land contained a few remnant tree species including *Prunus africana*.

**Figure 3:**
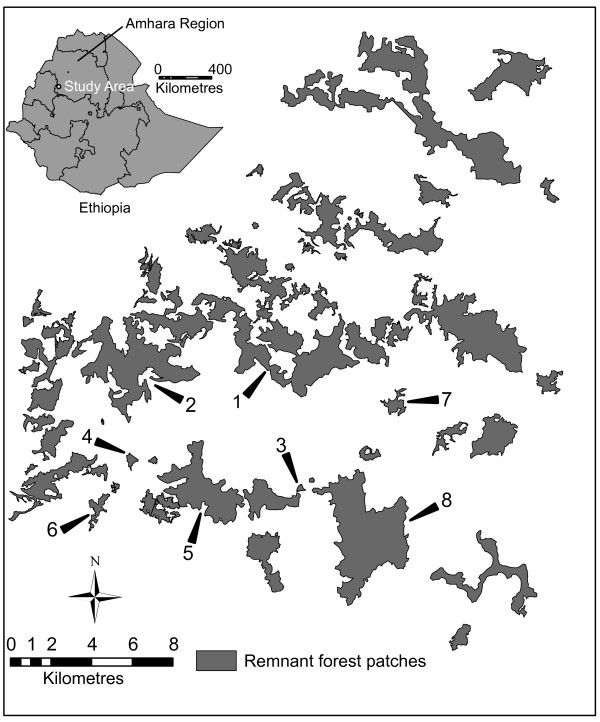
Location of study sites in NW Ethiopia.

Maps of the forest patches were digitized from high resolution satellite images of the study region. The digitized images were processed in Arc-Map10 (ESRI Inc.) to compute patch area and distance to the nearest patch (a measure of patch isolation). Following this, four large (650 -1389 ha) and four small (8 - 62 ha) remnant study patches were selected (see Figure 3). Nearest distances (edge to edge) for the large patches were between 4 m and 88 m except for one large patch, which was 291 m. Nearest distances for small patches were between 156 m and 1218 m. The unbalanced design for nearest distance was due to lack of the study species in some of the remnant forest patches. Pairwise geographical distances between centers of patches were estimated using the ‘distance measure’ facility in Arc-Map10 (ESRI Inc.). Estimated pair wise geographical distances among sampling sites ranged between 2.9 km and 15.5 km.

In each patch, *P. africana* trees and seedlings were sampled across a transect line of variable shape. To minimize the probability of sampling clonally propagated individuals, fruiting *P. africana* trees growing at least 20 m apart were selected for leaf or bark sample collection. Selected fruiting trees were coded and tagged using Aluminium tags. We used an eight meter tele-pole pruner to collect healthy leaf samples. Bark samples were collected where access to leaf samples was difficult. Two hundred and ten adult trees were processed. The circumference (at breast height, i.e. 1.3 m from ground) of each fruiting tree was measured using a measuring tape. Height was visually estimated. Where available, up to 6 seedlings (estimated height 5 cm to 30 cm) per fruiting tree were sampled near the canopy cover of selected fruiting individuals. Two hundred and thirty two seedlings were processed. Readings of elevation and geographical position were taken for each sampled fruiting tree and seedling. Leaf and bark samples were dried in locked plastic bags containing crystals of silica gel. Samples were stored in a -80°C freezer until required for DNA extraction.

### DNA extraction, amplification and genotyping

About 0.25 g of dried leaf and bark samples were machine-ground using a TissueLyser II and 3 mm tungsten beads (QIAGEN Inc.). The TissueLyser grinding was operated at a frequency of 30/s in two minute interval up to 7 times or until samples were finely powdered. Genomic DNA was extracted following a modified Cetyltrimethylammonium bromide (CTAB) protocol [65]. This protocol was slightly modified by applying a longer incubation period (for at least 8 hours) after adding and thoroughly mixing 750 μl of 2X CTAB buffer and 3μl of 2-mercaptoethanol to powdered samples in 1.5 ml Eppendrof tube.

Eight nuclear microsatellite DNA markers (EMPA001, EMPAS06, EMPA010, EMPAS10, EMPA016, PS12A02, EMPAS01 and UDP96-018) originally developed for other species of the same genus but later transferred to *P. africana*[66] were used to amplify DNA samples via polymerase chain reaction (PCR). A single PCR reaction (10 μl volume) consisted of: double distilled water, 10× buffer, 25 μM MgCl_2_, 10 μM dNTP, 10 μM forward primer, 10 μM reverse primer, 10 mg/ml BSA, 10 μM fluorescent-labelled M13(-21) primers (VIC, FAM, PET OR NED), 1 U/μl Red Taq DNA polymerase (PROMEGA) and about 10 ng of template DNA. The forward primer had the universal M13 tail where the labelled M13 primer can attach. Optimized PCR conditions consisted of a cycle of initial denaturing at 94°C for 5 minutes followed by 35 cycles of denaturing at 94°C for 30 s, annealing at 55°C for 45 s and extension at 72°C for 45 s. The final extension of amplicons was set at 72°C for 10 minutes. The PCR products were subjected to electrophoresis to isolate and size microsatellite fragments against an internal LIZ size standard (GeneScan500(-250)LIZ) on a 3130 Genetic Analayzer (APPLIED BIOSYSTEMS). Allele scoring was performed in GeneMapper 4.1 (APPLIED BIOSYSTEMS). Genotyped microsatellite data were tested (after 1000 randomizations) for occurrence of null alleles, stuttering or large allele dropout using MICRO-CHECKER version 2.2.3 [67]. These tests were crucial because errors introduced by null alleles, stuttering or large allele dropout may significantly bias population genetic estimates [68].

### Statistical analyses

All statistical analyses were performed separately for adults and seedlings as well as for the overall genotype data set consisting of both adults and seedlings. To assess deviation of genotypes from Hardy-Weinberg equilibrium, exact tests [69] were performed by the Markov chain method using the GENEPOP software version 4.0.10 [70] at the default analysis parameters (i.e., after 1000 dememorizations, 100 batches and 1000 iterations per batch). GENEPOP was also used to test for genotypic linkage disequilibrium for each pair of loci in each population using the log likelihood ratio statistic at the default values of the Markov chain parameters.

#### *Within population genetic diversity*

We determined unbiased genetic diversity parameters (allelic richness and gene diversity) for each population at 7 polymorphic microsatellite markers as implemented in FSTAT version 2.9.3.2 [71]. Allelic richness per locus and population was based on a minimum sample size of 16, 7 and 6 diploid individuals in the overall, adult and seedling populations, respectively. FSTAT was also used to test for differences in genetic diversity between populations from two forest patch size groups i.e. large (n = 4) vs small (n = 4). The p-values in these analyses (two-tailed tests) were obtained after 15000 permutations. Average allelic richness values per population were computed across the 7 loci and tested using linear regression analysis in SAS 9.2 (SAS Institute Inc.) against nearest distance (a measure of forest patch isolation) or forest patch size. Both environmental variables were log_10_ transformed prior to analysis to approximate normality. To meet the basic assumptions for the linear regression analyses we always performed residual diagnostics for normality, homoscedasticity, linearity and independence problems. Fortunately, we did not encounter any problem regarding these assumptions. The level of variance in genetic diversity based on allelic richness among the two patch size (i.e., large vs small) and patch isolation (less-isolated vs isolated) groups was also evaluated using the Student’s t-test in SAS 9.2 (SAS Institute Inc.). Patch size and isolation groupings were made using median values for size and isolation. The existence of inbreeding was evaluated by comparing both the inbreeding coefficient (*F*_IS_) and corrected genetic relatedness (Relc) among the two population groups as implemented in FSTAT. Mean allelic richness, gene diversity, number of alleles and inbreeding coefficient (*F*_IS_) were compared between cohorts using paired t-test in SAS 9.2 (SAS Institute Inc.).

#### *Population differentiation and genetic structure analyses*

Population differentiation and extent of genetic structuring was evaluated by computing pairwise population genetic distances (*F*_ST_) and testing for significance after 10,100 permutations as implemented in ARLEQUIN version 3.5.1.2 [72]. Average population-specific *F*_ST_ values were compared between cohorts using paired t-test and between patch size and isolation groups using the Student’s t-test in SAS 9.2 for the overall data set. The mean population-specific *F*_ST_ values (see below) were also linearly regressed against patch size or isolation in SAS 9.2 based on the overall data set. The spatial pattern of genetic differentiation among remnant forest patches was assessed by running a Mantel test [73] in ARLEQUIN. In this test 100,000 permutations were performed to evaluate significance of the correlation between matrices of the genetic (pair wise *F*_ST_) and geographic distances (point distance measured in Km between centres of patches) between pairs of populations. Partial Mantel tests were performed in ARLEQUIN applying 100,000 permutations to check the correlation of matrices of the genetic and two environmental variables (patch size and geographical or patch nearest distances) while keeping constant one of these environmental variables (patch size or pairwise geographical distance between centres of sampled patches or patch nearest distance). Spatial genetic patterns were also assessed by applying a hierarchical Bayesian method to compute population-specific *F*_ST_ values and relate them to environmental variables through a generalized linear model in GESTE version 2.0 [74]. All parameters for this analysis were set to default values. Environmental variables tested in this analysis include patch nearest distance, patch size and mean pairwise geographical distances between centers of sampled patches.

Standardized *F*_*ST*_ (*F’*_ST_) and Jost’s D_est_[55,56] were computed using RecodeData v.0.1 [56] and GeneAlex 6.5 [75,76] to minimize the dependence of *F*_*ST*_ on the genetic diversity of cohorts. Computation of these estimates was necessary because uncorrected *F*_ST_ comparison of seedlings and adults could be compromised by differences in their genetic diversity. Tests for recent reduction in effective population size were carried out in Bottleneck version 1.2.02 [49]. These analyses were performed by applying 10,000 simulations under the two-phase mutation model (TPM) with a 95% stepwise and 5% multi-step mutations, and 12% variance for the multiple steps as suggested for microsatellite data [49]. Because the numbers of loci employed in this study were fewer than recommended for Bottleneck analyses [47], statistical significance was based on the Wilcoxon’s signed-rank test for heterozygosity excess. Lastly, analysis for detection of first generation migrants was performed in Geneclass2 [52] using the ‘likelihood computation L = L_home/L_max’. The criterion for this computation was based on a Bayesian method [77]. Ten thousand simulations [78] were run setting a threshold *p*-value of 0.01.

## Abbreviations

NW: North-western; IBD: Isolation by distance; P: *africana*: *Prunus africana*; CTAB: Cetyltrimethylammonium bromide; PCR: Polymerase chain reaction; Km: Kilometer; Ne: Effective population size; TPM: Two-phase mutation model; IBC: Institute of biodiversity conservation; DAFF: Department of agriculture, fisheries and forestry; Hs: Gene diversity; FIS: Inbreeding coefficient; MNA: Mean number of alleles; CITES: Convention on international trade in endangered species of wild fauna and Flora; IUCN: International union for conservation of nature; ICRAF: World agroforestry centre.

## Competing interests

Authors declare that they have no competing interests.

## Authors’ contributions

HY conceptualized, designed and conducted the field and laboratory works, analyzed data, and drafted the manuscript. DS conceptualized this work, guided in data analyses and critically improved the draft manuscript for intellectual content. JH conceptualized and designed the study, guided and commented all data analyses and critically improved the draft manuscript for intellectual content. All authors read and approved the final manuscript.

## Supplementary Material

Additional file 1 **We show in this table environmental variables and genetic diversity estimates of *Prunus africana *** **in NW Ethiopia based on the overall data set.** Click here for file

Additional file 2 **In this file we illustrate linear regression of fragmentation variables against allelic richness and population-specific *F***_**ST **_**of *Prunus africana *** **in NW Ethiopia (Note: Area and isolation were Log10 transformed; results of all the linear regression tests were non-significant).** Click here for file

Additional file 3 Here the results of the Wilcoxon’s test for population bottlenecks under TPM and SMM are provided. Click here for file

Additional file 4 **This table shows sample size and proportion of first generation migrants in the seedling and adult populations of *P. africana *** **in NW Ethiopia.** Click here for file

Additional file 5 **This photographic file demonstrates human impact and the occurrence of extensive logging and felling of age-old *P. africana *** **trees in the remnant forest patches of NW Ethiopia.** Click here for file
