# Supplementary material for: Genetic structuring of remnant forest patches in an endangered medicinal tree in North-western Ethiopia
Source: BMC Genet. 2014 Mar 6;15:31. doi: 10.1186/1471-2156-15-31 (PMC4021171; doi:10.1186/1471-2156-15-31)
Supplement: Additional file 1 — We show in this table environmental variables and genetic diversity estimates of Prunus africana in NW Ethiopia based on the overall data set. [file 1471-2156-15-31-S1.pdf]

Additional File 1. Environmental variables and genetic diversity estimates based on the overall data set (Note: Near_dist = patch isolation in meters, patch_size = patch area in sq.m, PSC = Patch size class, PIC = Patch isolation class, L = Large, S = Small, C = Less-isolated, I = Isolated, AR = Allelic richness, HS = Gene diversity, F_{IS} = Inbreeding coefficient, MNA = Mean number of alleles).

**Population**	Near_dist	Patch_size	Altitude	Latitude	Longitude	**PSC**	**PIC**	**AR**	**HS**	**F_{IS}**	**MNA**
Bradi	24	11777280	2139	10.85	36.60	L	C	8.437	0.783	-0.097	12.143
DarabaSigsi	89	10616691	2031	10.84	36.54	L	C	8.674	0.775	0.143	11.286
Demba	57	77514	2134	10.79	36.62	S	C	7.755	0.762	-0.123	11.000
Dishi	479	201586	2073	10.81	36.54	S	I	7.886	0.753	0.022	11.571
Kambo	4	6498332	2054	10.80	36.57	L	C	8.482	0.761	0.053	11.857
Metin	156	588037	1997	10.79	36.52	S	I	6.778	0.698	0.007	7.000
Temcha	1218	621111	2268	10.81	34.65	S	I	7.337	0.726	-0.036	10.143
Wonse	292	13887144	2030	10.77	36.63	L	I	7.909	0.749	-0.046	10.000
