# Supplementary material for: Genetic structuring of remnant forest patches in an endangered medicinal tree in North-western Ethiopia
Source: BMC Genet. 2014 Mar 6;15:31. doi: 10.1186/1471-2156-15-31 (PMC4021171; doi:10.1186/1471-2156-15-31)
Supplement: Additional file 2 — In this file we illustrate linear regression of fragmentation variables against allelic richness and population-specific F ST of Prunus africana in NW Ethiopia (Note: Area and isolation were Log10 transformed; results of all the linear regression tests were non-significant). [file 1471-2156-15-31-S2.pdf]

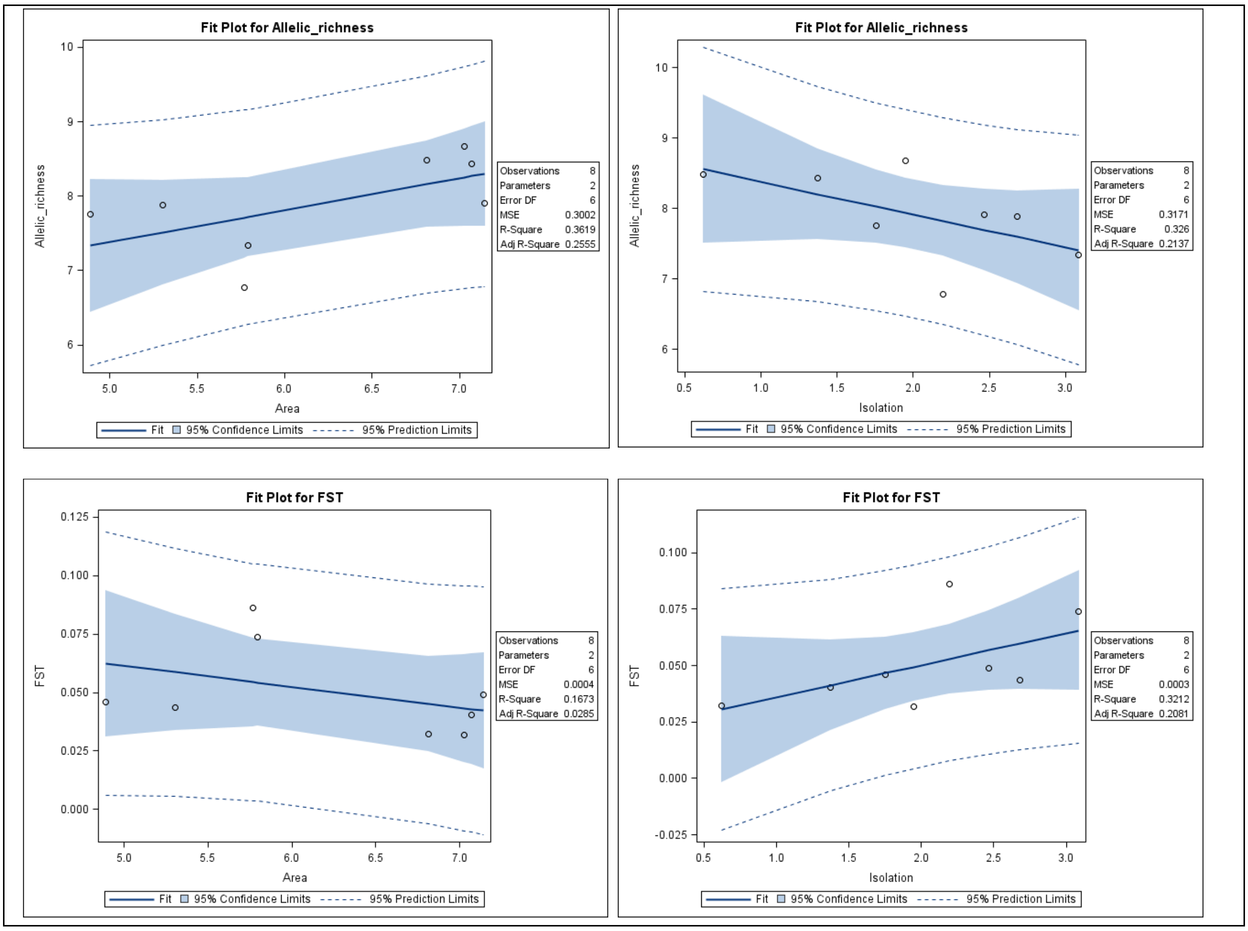

Additional File 2. Linear regression of fragmentation variables against allelic richness and population-specific F_{ST} (Note: Area and isolation were Log10 transformed; results of all the linear regression tests were non-significant).
