# Supplementary material for: Genetic structuring of remnant forest patches in an endangered medicinal tree in North-western Ethiopia
Source: BMC Genet. 2014 Mar 6;15:31. doi: 10.1186/1471-2156-15-31 (PMC4021171; doi:10.1186/1471-2156-15-31)
Supplement: Additional file 3 — Here the results of the Wilcoxon’s test for population bottlenecks under TPM and SMM are provided. [file 1471-2156-15-31-S3.pdf]

Additional File 3. Wilcoxon's signed-rank tests for population bottlenecks (Probability for H excess after 10000 replications. Parameters for T.P.M.: Variance = 12% Probability = 95%) (Note: L = large, C = less-isolated; TPM = Two- Phase Model; SMM = Stepwise Mutation Model)

Population	Overall		Adults		Seedlings	
	TPM	SMM	TPM	SMM	TPM	SMM
Bradi (L, C)	0.988	1.00	0.594	0.594	1.000	1.000
DarabaSigsi (L, C)	0.945	0.988	0.945	0.961	0.344	0.656
Demba (S, C)	0.992	0.996	0.980	0.980	0.656	0.961
Dishi (S, I)	0.988	0.996	0.148	0.594	0.973	0.980
Kambo (L, C)	0.988	1.000	0.988	1.000	0.711	0.945
Metin (S, I)	0.945	0.980	0.148	0.289	0.711	0.711
Temcha (S, I)	1.000	1.000	0.656	0.813	0.945	0.980
Wonse (L, I)	0.973	0.988	0.766	0.852	0.988	0.992
