# Supplementary material for: Genetic structuring of remnant forest patches in an endangered medicinal tree in North-western Ethiopia
Source: BMC Genet. 2014 Mar 6;15:31. doi: 10.1186/1471-2156-15-31 (PMC4021171; doi:10.1186/1471-2156-15-31)
Supplement: Additional file 4 — This table shows sample size and proportion of first generation migrants in the seedling and adult populations of P. africana in NW Ethiopia. [file 1471-2156-15-31-S4.pdf]

Additional File 4. Sample size and proportion of first generation migrants (Note: the column '% Migrant seedling' represents analysis based on the overall data set; L = large; C = less-isolated).

Population	Number of individuals		Grand Total	% Migrant Adult	% Migrant Seedling
	Adult	Seedling			
Bradi (L, C)	26	36	62	7.692	8.333
DarabaSigsi (L, C)	29	20	49	6.897	10.000
Demba (S, C)	25	30	55	8.000	10.000
Dishi (S, I)	33	49	82	6.061	6.122
Kambo (L, C)	44	28	72	2.273	7.143
Metin (S, I)	12	10	22	0.000	10.000
Temcha (S, I)	24	30	54	0.000	6.667
Wonse (L, I)	17	29	46	5.882	6.897
**Grand Total**	**210**	**232**	**442**		
