# Supplementary figures and images for: Genetic structuring of remnant forest patches in an endangered medicinal tree in North-western Ethiopia
Source: BMC Genet. 2014 Mar 6;15:31. doi: 10.1186/1471-2156-15-31 (PMC4021171; doi:10.1186/1471-2156-15-31)

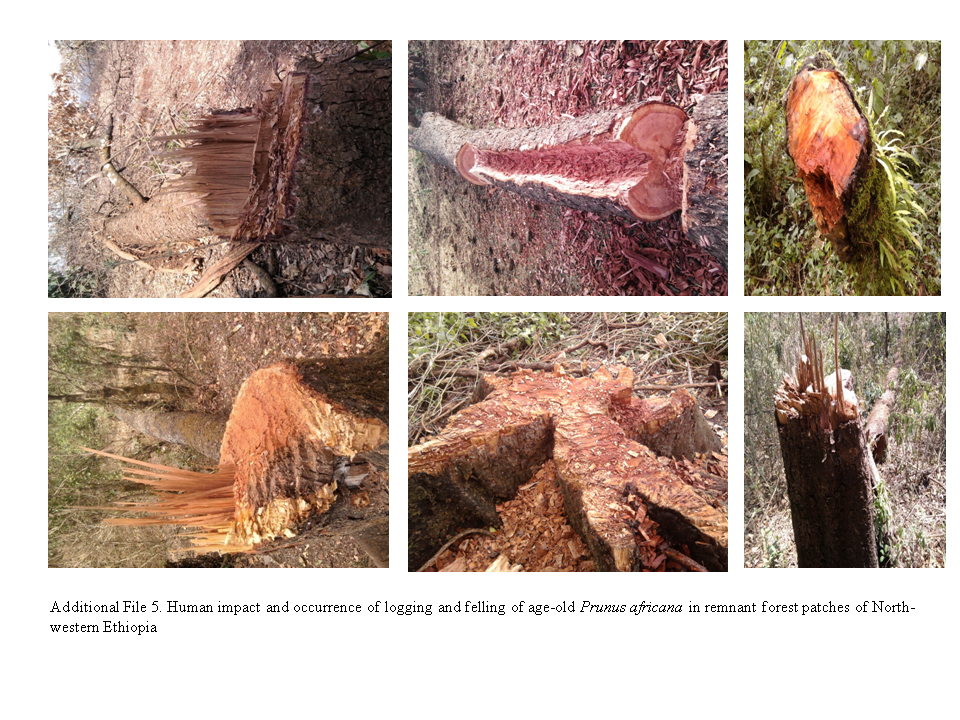

Supplement: Additional file 5 — This photographic file demonstrates human impact and the occurrence of extensive logging and felling of age-old P. africana trees in the remnant forest patches of NW Ethiopia. [file 1471-2156-15-31-S5.tiff]
